# Efficacy of Prosopilosidine from *Prosopis glandulosa* var. *glandulosa* against *Cryptococcus neoformans* Infection in a Murine Model

**DOI:** 10.3390/molecules23071674

**Published:** 2018-07-10

**Authors:** Mohammad K. Ashfaq, Mohamed Sadek Abdel-Bakky, Mir Tahir Maqbool, Volodymyr Samoylenko, Aziz Abdur Rahman, Ilias Muhammad

**Affiliations:** 1 National Center for Natural Product Research, Research Institute of Pharmaceutical Sciences, School of Pharmacy, University of Mississippi, University, MS 38677, USA; abdelbakkym@yahoo.com (M.S.A.-B.); tmmir@olemiss.edu (M.T.M.); voxa@outlook.com (V.S.); aziz2002@ru.ac.bd (A.A.R.); 2 Faculty of Pharmacy, Al-Azhar University, Cairo 11651, Egypt; 3 Department of Arts and Sciences, Keiser University, 2085 Vista Pkwy, West Palm Beach, FL 33411, USA; 4 Department of Pharmacy, University of Rajshahi, Rajshahi 6205, Bangladesh

**Keywords:** *Cryptococcus neoformans*, cryptococcosis, HepG2, *Prosopis glandulosa*, prosopilosidine, amphotericin B, fluconazole

## Abstract

In this study, 2,3-dihydro-1*H*-indolizinium alkaloid-prosopilosidine (PPD), that was isolated from *Prosopis glandulosa*, was evaluated against *C. neoformans* in a murine model of cryptococcosis. In vitro and in vivo toxicity of indolizidines were also evaluated. Mice were infected via the tail vein with live *C. neoformans*. Twenty-four hours post-infection, the mice were treated with PPD once a day (i.p.) or twice a day (*bid*) orally, or with amphotericin B (Amp B) intraperitoneally (IP), or with fluconazole (Flu) orally for 5 days. The brains of all of the animals were aseptically removed and the numbers of live *C. neoformans* were recovered. In vitro toxicity of indolizidine alkaloids was determined in HepG2 cells. PPD showed to be potent in vivo activity against *C. neoformans* at a dose of 0.0625 mg/kg by eliminating ~76% of the organisms compared to ~83% with Amp B (1.5 mg/kg). In addition, PPD was found to be equally efficacious, but less toxic, at either 0.125 or 0.0625 mg/kg compared to Amp B (1.5 mg/kg) when it was administered *bid* (twice a day) by an i.p. route. When tested by an oral route, PPD (10 mg/kg) showed potent activity in this murine model of cryptococcosis with ~82% of organisms eliminated from the brain tissue, whereas Flu (15 mg/kg) reduced ~90% of the infection. In vitro results suggest that quaternary indolizidines were less toxic as compared to those of tertiary bases. PPD (20 mg/kg) did not cause any alteration in the plasma chemistry profiles. These results indicated that PPD was active in eliminating cryptococcal infection by oral and i.p. routes at lower doses compared to Amp B. or Flu.

## 1. Introduction

*Cryptococcus neoformans* is a dimorphic fungus that causes serious infection leading to pneumonia and life-threatening diseases of the central nervous system (CNS). The virulence of the organism has been linked to the presence of a thick carbohydrate capsule and its pigment melanin [1,2]. Both immunocompetent and immunocompromised individuals are affected; immunocompetent patients generally develop pulmonary cryptococcosis as a sole manifestation of the disease [3]. However, in immune compromised and elderly patients, both lung and brain infections cause high morbidity and mortality [4,5]. In advanced cases of HIV infections, the incidence of cryptococcal infection ranged from 10–15% in developed countries and was even higher in the underdeveloped countries [6,7]. With the advent of antiretroviral therapy (ART), the immune system of patients with HIV/AIDS became less vulnerable to fungal infections or other infections, and this reduced the incidence of *C. neoformans* infections in people with advanced HIV/AIDS, especially those from developed countries [8]. Thus, fungal infections in HIV/AIDS patients declined significantly in the U.S. [9,10]. However, it still remains a major problem in developing countries due to limited healthcare facilities [11]. It is estimated that globally, cryptococcal meningitis is responsible for 15% of AIDS-related deaths, with an estimate of 220,000 new cases of cryptococcal meningitis each year, resulting in 181,000 deaths [11]. Hence, cryptococcosis still remains a concern for people living with HIV/AIDS.

Only a limited number of antifungals are available for the treatment of Cryptococcal infections. Amphotericin B (Amp B) and fluconazole (Flu) are among the commonly used antifungals for patients with cryptococcal infections of the CNS. However, the emergence of Flu-resistant fungal pathogens is a concern [12]. In vitro investigations on various strains of *C. neoformans* indicate that mutation to Flu resistance is a dynamic and heterogeneous process that involves multiple mechanisms [13,14]. Recently, concerns have been raised about the efficacy of initial Flu treatments of cryptococcal meningitis in HIV patients, as resistance to Flu leads to a relapse of cryptococcal meningitis symptoms [15,16]. In such cases, a combination of Amp B and Flu or voriconazole has been suggested [17,18].

According to World Health Organization (WHO) guidelines, the treatment for cryptococcal meningitis should be comprised of a combination of therapies, starting with Amp B and flucytosine for 2-weeks, followed by Flu [17,18]. All of these anti-fungal agents have different mechanisms of action against Cryptococcal infections. Amp B makes the yeast membranes porous by binding with ergosterol [19], flucytosine prevents protein synthesis by intercalating into fungal RNA [20,21], and Flu acts by binding and inhibiting 14-α demethylase- enzyme which is important for ergosterol synthesis in fungal cells [22]. Combinational therapy of these drugs with different mechanisms of action makes it difficult for fungal cells to develop resistance against these drugs during the course of treatment [23]. However, flucytosine is associated with bone marrow suppression [24]. Also, flucytosine is excessively expensive (>$500/day) and is not licensed in many countries [25]. Therefore, WHO recommended the use of Flu in place of flucytosine [17]. The emergence of resistance to Flu and the toxicity of Amp B [26,27] underscore the need to search for new compounds that have anti-cryptococcal activity.

**Figure 1:**
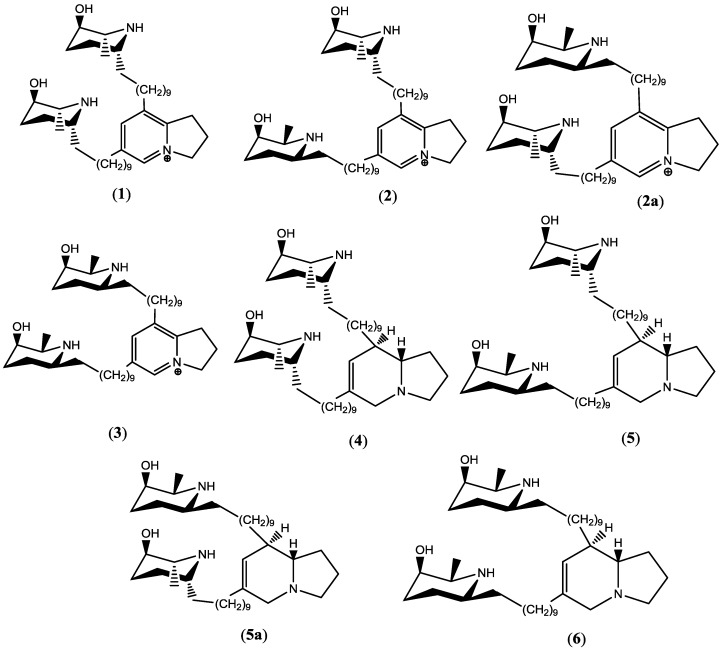
Chemical structures of indolizidine alkaloids isolated from *P. glandulosa* var. *gladulosa*: prosopilosidine (**1**), isoprosopilosidine (**2**/**2a**), juliprosine (**3**), prosopilosine (**4**), isoprosopilosine (**5**/**5a**), and juliprosopine (**6**).

Natural products have played a significant role in building the armory of anti-infectives. However, natural products of plant origin with anti-infective property have rarely been introduced as drug(s). *Prosopis glandulosa*, a medium-sized tree, is one of the two varieties of honey mesquites that is available in North America [28,29]. The genus *Prosopis* contains indolizidine alkaloids substituted with alkyl piperidine unit(s), of which juliprosopine exhibited strong in vitro antimicrobial, anti-dermatophytic, and amebicidal activities [30,31]. In continuation to our earlier work on indolizidine alkaloids [32], herein we report the in vivo anticryptococcal activity of PPD, which was isolated from *Prosopis glandulosa*, in a murine model. In addition, we also report the in vitro toxicity on HepG2 cells of indolizidine alkaloids (Prosopilosidine (**1**), isoprosopilosidine (**2**), juliprosine (**3**), prosopilosine (**4**), isoprosopilosine (**5**), and juliprosopine (**6**) (Figure 1) which were isolated from *P. glandulosa* that was collected from Nevada and Texas, USA.

## 2. Results and Discussion

### 2.1. In Vitro Toxicity of ***1***–***6*** against HepG2 Cells

**Figure 2:**
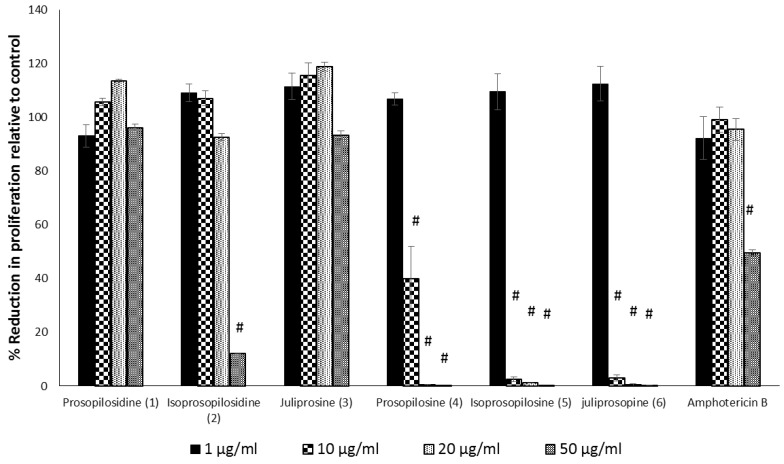
The effect of different compounds, prosopilosidine (**1**), isoprosopilosidine (**2**), juliprosine (**3**), prosopilosine (**4**), isoprosopilosine (**5**), juliprosopine (**6**) and Amphotericin B (Amp B) on cell viability. HepG2 cells were seeded on 96-well plates and were treated with 1, 10, 20, and 50 µg/mL of the test compounds or with vehicle only for 24 h. The MTS assay for cell viability and proliferation was performed with the CellTiter 96^®^AQueous Non-Radioactive Cell Proliferation Assay Kit (Promega). Data are shown as mean ± SEM and were analyzed with ANOVA. ^#^ *p* < 0.001 is considered statistically significant compared to the untreated cells (100%).

In the MTS assay (Abcam, Cambridge, MA, USA), the treatment of HepG2 with tertiary indolizidine alkaloids, prosopilosine (**4**), isoprosopilosine (**5**), and juliprosopine (**6**) were found to be toxic at a concentration of >1 µg/mL. On the other hand, the two quaternary indolizidines, PPD and juliprosine (**3**), both with symmetrical piperidenyl side chains, did not show any toxicity up to 50 µg/mL, while their non-symmetrical diasterioisomer isoprosopilosidine (**2**) was found to be toxic at 50 µg/mL (Figure 2). Collectively, the evaluation of the cytotoxicity of indolizidine alkaloids against HepG2 cells revealed that the quaternary PPD and juliprosine (**3**) were found to be less toxic to those of tertiary indolizidine alkaloids (**4**–**6**), a phenomenon that is consistent with their toxicities against VERO cells (Samoylenko et al., 2009) [29].

### 2.2. In Vivo Anti-Cryptococcal Activity of PPD

Two 2,3-dihydro-1*H*-indolizinium alkaloids, PPD and isoprosopilosidine (**2**), which were isolated from *P. glandulosa*, have demonstrated potent in vitro antifungal activity against *C. neoformans* and antibacterial activity against methicillin-resistant *S. aureus* and *M. intracellulare* [32]. Due to its potent in vitro activity against *C. neoformans* and its lower toxicity in mammalian VERO cells [32], anti-cryptococcal activity was conducted on PPD in vivo. The maximum tolerated i.p. dose in mice was determined to be 2.5 mg/kg. At this dose, no deaths were observed by the intraperitoneal route. At 5.0 and 10.0 mg/kg, deaths were observed (Table 1).

**Figure 3:**
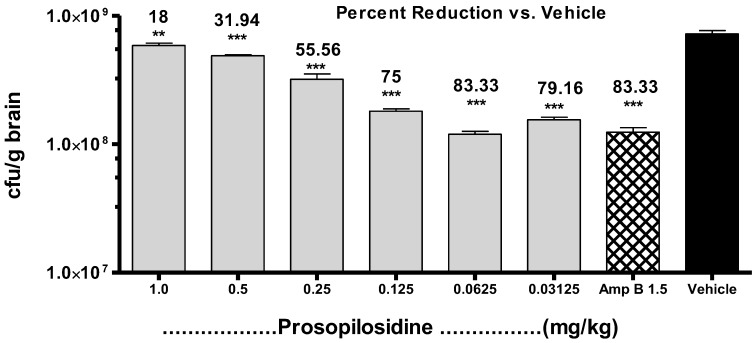
Treatment of *Cryptococcus neoformans* infection with different doses of prosopilosidine administered for 5 days in a murine model. Graph showing live colony forming units (CFU) of *C. neoformans* per gram of brain tissue with percent reduction in infection by prosopilosidine treatment given at doses 0.03125, 0.06215, 0.125, 0.25, 0.5, and 1.0 mg/kg body weight as compared to the vehicle control. Amp B: Amphotericin B (1.5 mg/kg body weight). Data are shown as mean + SEM (*n* = 5) and was analyzed with ANOVA. ** *p* < 0.01, *** *p* < 0.001 is considered statistically significant compared to the vehicle control.

Based on the MTD results, an exploratory in vivo experiment to determine the anti-cryptococcal activity of PPD by i.p. route was performed in a rodent model. In this exploratory experiment, treatment with PPD showed a dose dependent effect at low doses. In order to determine the maximum anti-cryptococcal effect of PPD, additional experiments were conducted with the addition of Amp B as a reference standard (1.5 mg/kg; i.p.), with doses ranging between 0.125 and 1.0 mg/kg/day for 5 days. PPD showed potent activity against *C. neoformans* at 0.125 mg/kg/day, with ~75% of organisms eliminated from the brain tissue, whereas Amp B at 1.5 mg/kg/day reduced ~83% of infection. Thus, the dose-dependent antifungal effect at lower doses, as was observed in our preliminary study, was reconfirmed in this experiment. Finally, the lowest dose for the maximum inhibitory effect, together with the dose-response relationships, were determined by another in vivo anti-cryptococcal experiment with 4 doses of 0.03125, 0.0625, 0.125, and 0.25 mg/kg/day. The maximum activity was observed at 0.0625 mg/kg, followed by 0.125 mg/kg, 0.25 mg/kg, 0.03125 mg/kg, where 80, 76, 71, and 68% of the organisms were eliminated from the brain tissue, respectively, vs. 83% by Amp B (Figure 3). However, increasing the dose further to 0.125 mg/kg/day or decreasing it to 0.03125 mg/kg/day did not change the antifungal activity significantly.

The i.p. administration of PPD showed increasing activity against *C. neoformans* with decreasing concentration. This phenomenon appears to be similar to the “hormesis hypothesis” which was reported earlier [33,34], where high doses of a compound showed no observable effect, while at low doses, the effect was observed. It is unclear from the present study what could be the cause for this phenomenon. However, explanations can be speculated. It has been reported that certain compounds at different dose levels can induce different CYP enzymes in mice; at low doses (<0.25 mg/kg), perfluorodecanoic acid (PFDA) increases Cyp4A14 expression. Whereas, at higher doses (>10 mg/kg), in addition to CYP4A14, PFDA elevates the expression of Cyp2B10 [35]. It is possible that in our study, the i.p. administration of PPD at higher doses induced a set of CYP enzymes that metabolized it and rendered it ineffective against *C. neoformans*. At a low dose, such enzymes may not be induced and thus, a beneficial effect was observed at low doses. Another possibility lies in the transport of the compound inside the fungal cells. The channels by which this compound reaches the cryptococcal cells may be blocked at higher doses. It is known that many compounds up or down regulate different genes at different dose levels in yeast organisms [35]. One or more of the above hypothetical explanations may be responsible for the hormesis phenomenon that was observed in this study.

### 2.3. Bid Treatment with PPD 

**Figure 4:**
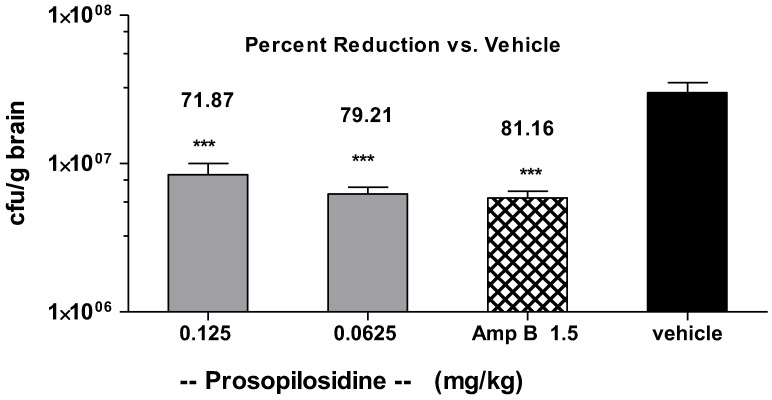
*Cryptococcus neoformans* infection in the mice treated with prosopilosidine administered *bid*. Graph showing live colony forming units (CFU) of *C. neoformans* per gram of brain tissue and percent reduction in infection by prosopilosidine treatment given at doses 0.06215 and 0.125 mg/kg body weight and amphotericin B (1.5 mg/kg body weight), as compared to the vehicle control. Amp B: Amphotericin B (1.5 mg/kg body weight). Data are shown as mean + SEM (*n* = 5) and was analyzed with ANOVA. *** *p* < 0.001 was considered statistically significant compared to the vehicle control.

In an attempt to see if two doses per day would increase the antifungal effect of PPD, we considered *bid* (twice a day) in vivo treatment. Mice were infected via the i.v. route on day 0 and were then treated with PPD (24 h post infection) via the intraperitoneal route twice a day for 5 days. Figure 4 shows the comparison of PPD and Amp B in the mice. The number of *C. neoformans* organisms was substantially reduced in the brain by a *bid* dose of 0.0625 or 0.125 mg/kg. At these doses, the infection was reduced by 79.21% and 71.87%, respectively, with Amp B showing a 81.16% reduction in the number of *Cryptococcus* organisms in the brain tissue. It is important to mention that none of the animals that were treated with PPD showed any signs of distress during this dose regimen. Amp B treated animals showed rough hair coat throughout the course of the treatment, which is indicative of stress. The vehicle control group did not show any other sign of distress.

### 2.4. Oral Administration of PPD

In a separate set of experiments, we attempted oral administration of PPD. These mice did not show any signs of distress for up to 4 h after PPD administration via the oral route. Then, the animals were sacrificed by CO_2_ asphyxiation. At necropsy, all visceral organs appeared to be normal. No remarkable lesion was seen in the GI tract on gross examination. The blood clinical chemistry showed no shift in any of the parameters listed in Table 2.

**Figure 5:**
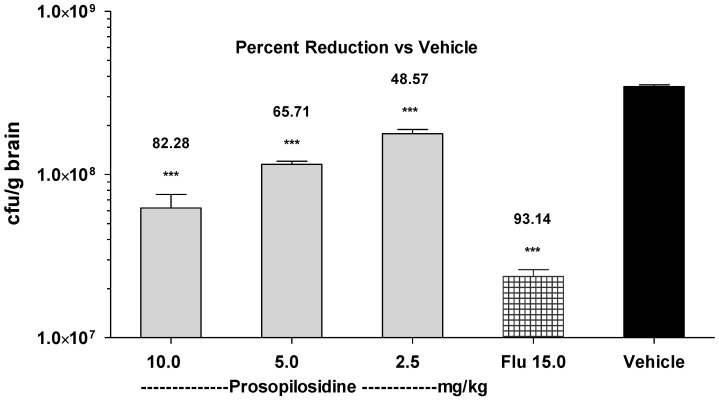
*Cryptococcus neoformans* infection in mice treated with prosopilosidine given via the oral route. Graph showing live colony forming units (CFU) of *C. neoformans* per gram of brain tissue and the percent reduction in infection by prosopilosidine treatment given at doses 2.5, 5.0, and 10.0 mg/kg body weight and Fluconazole (Flu) (15 mg/kg body weight) as compared to the vehicle control. Data are shown as mean ± SEM (*n* = 5) and was analyzed with ANOVA. *** *p* < 0.001 was considered significant as compared to the vehicle control.

This data confirmed that PPD was not toxic if given orally up to 20 mg/kg. We then conducted an efficacy study with the oral administration of PPD in the infected mice. The mice were infected by *Cryptococcus neoformans* inoculum via the i.v. route on day 0 and were then treated with PPD (24 h post infection) via the oral route once a day for 5 days. Oral administration of PPD showed a dose dependent activity against *C. neoformans*. Figure 5 shows the number of live organisms (CFU) that were recovered from the brains of the mice who were treated with PPD (using doses of 2.5, 5.0, and 10 mg/kg/day), Flu (15 mg/kg/day), or the vehicle. PPD, Flu, and the vehicle were administered orally in a volume of 100 μL per mouse via oral gavage needle. The vehicle treated group showed an average of 3.5 × 10^8^ CFUs in their brain tissues. In comparison, orally administered Flu at 15 mg/kg eliminated the organism in the brain, showing over 93% reduction in infection compared to the vehicle control. At doses 2.5, 5.0, and 10 mg/kg, a direct dose-effect relationship was observed with a maximum 82% reduction in infection which was observed at 10 mg/kg. At doses 2.5 and 5.0 mg/kg, the reduction in infection was 48% and 65%, respectively. It is evident from these results that PPD reached the target organ after absorption and was able to reduce the *C. neoformans* load in the brain tissue. It is also clear from these results that PPD was well tolerated, since no deaths or apparent signs of toxicity were observed.

## 3. Materials and Methods

### 3.1. Compounds and Chemicals

Prosopilosidine (**1**), isoprosopilosidine (**2**), prosopilosine (**4**), isoprosopilosine (**5**), and juliprosopine (**6**) were isolated from *P. glandulosa* (Fam. Leguminosae), which was collected from Nevada, USA, as described previously by Samoylenko et al. (2009) [29]. In addition, compounds (**3**) and (**6**) were isolated from the *P. glandulosa* sample that was collected in Texas. Amp B and Flu were purchased from Sigma-Aldrich (St. Louis, MO, USA). Amphotericin B and PPD were separately dissolved as a stock solution (1 mg/mL containing 13.3 µL DMSO, 13.3 µL Tween-20, and 973.4 µL distilled water) as colored and clear solutions, respectively. They were protected from light and were stored in the refrigerator. Different doses were prepared from this stock and were used daily. CellTiter 96^®^ AQueous Non-Radioactive Cell Proliferation Assay kit (MTS) was purchased from Promega (Madison, WI, USA).

### 3.2. Animals

Female mice (CD-1) weighing 20–25g were obtained from Envigo (Indianapolis, IN, USA). They were quarantined on arrival for at least 3 days at the University of Mississippi vivarium. All of the animals were housed in plastic cages with fiberglass filter tops and were provided with food and water *ad-libitum*. They were maintained according to the Institutional Animal Care and Use Committee (IACUC) guidelines of the University. All experiments reported here were approved by the IACUC protocol number 07-011.

### 3.3. Inoculum

*C. neoformans* ATCC 90113 was grown on Saburaud Dextrose Agar (SDA) at 30 °C for 48 h to check for purity. A single colony was inoculated to 20 mL Saburaud Dextrose Broth (SDB) in a 100 mL flask and was kept at 30 °C in a shaker incubator overnight. The broth culture was centrifuged in a 50 mL tube and was washed three times with PBS. The pellet was suspended in 10 mL PBS and was adjusted to approximately 1–5 × 10^6^ fungal cells/mL. This inoculum was kept on ice until all of the animal inoculations were completed. Serial dilutions from the inoculum were grown on SDA at 30 °C to confirm the inoculum size by determining the live colony forming units (CFU).

### 3.4. In Vitro Cytotoxicity Assay

Human hepatoma (HepG2) cells were maintained at 37 °C in equilibration with 5% CO_2_-95% air in 75-cm^2^ flasks containing maintenance medium plus 10% fetal bovine serum (FBS). The maintenance media (DMEM) consisted of 10% fetal bovine albumin, 1% nonessential amino acids, 1% l-glutamine and 100 U/mL penicillin, and 10 mg/mL streptomycin. Subcultures of the cells for use in the experiments were obtained from a 1:4 split of the confluent monolayers. The cells were seeded on a flat bottom 96 well plate (2 × 10^4^ cells per well). The cells were exposed to different doses of compounds **1**–**6** and tryptamine for 24 h, and cytotoxicity was determined using MTS assay kit according to the manufacturer’s instructions (Promega, Madison, WI, USA). Tryptamine was evaluated as a control, which was found to be non-toxic up to 50 μg/mL.

### 3.5. Maximum Tolerated Dose

The maximum tolerated dose (MTD) in the mice was determined by giving intraperitoneal (i.p.) injections of PPD, with doses ranging from 1 to 10 mg/kg, daily for 5 days. The body weights of the mice and their deaths were recorded. Necropsy was performed on all mice and gross changes were recorded. In a separate experiment, the mice were administered PPD orally at 20 mg/kg using oral gavage needles. Blood was collected 4 h later and was subjected to diagnostic profile Vetscan2 (Abaxis, Union City, CA, USA) to observe any change in the clinical chemistry parameters.

### 3.6. Experimental Design

Mice were inoculated with 100 μL of the inoculum intravenously (i.v.) via the tail vein and were distributed in various groups (*n* = 5/group). Twenty-four hours post inoculation, the mice were administered their respective treatments (different doses of PPD, Amp B, and vehicle) i.p. or orally using oral gavage needles for 5 days. The animals were dosed either once a day or twice a day at 12 h intervals (*bid*). On day 6 post inoculation, all mice were sacrificed by CO_2_ asphyxiation, as approved by the IACUC. Their brains were aseptically removed, weighed, and homogenized in 5 mL of PBS. Serial dilutions of these homogenates were made in PBS and were cultured in duplicates on SDA. After 48 h of incubation at 30 °C, CFU from each homogenate was enumerated. The number of CFU of *C. neoformans* per gram of brain tissue was determined for each mouse. Percent reduction in CFU’s per gram of brain tissue was calculated by formula:

$$\%\;\mathrm{reduction}=\frac{\mathrm{Vehicle}-\mathrm{Sample}}{\mathrm{Vehicle}}\times 100$$

### 3.7. In Vivo Clinical Chemistry of PPD

In order to assess the toxic potential of PPD by the oral route, a dose of 20 mg/kg (twice the maximum oral anti-cryptococcal dose used in this study) was given to mice (*n* = 5). Blood was sampled 4 h after the oral dose and liver profiles were determined by using VetScan2 (Abaxis, Union City, CA, USA). The major parameters included albumin, alanine transaminase (ALT), Blood urea nitrogen (BUN), glucose, total bilirubin, total protein, globulin, and electrolytes. Blood from three mice from each group was analyzed for clinical chemistry.

### 3.8. Statistical Analysis

Differences between the groups were analyzed using ANOVA followed by Dunnett’s multiple comparisons test using Graph Pad Prism 5.0, and the minimum criterion for statistical significance was set at *p* < 0.05 for all of the comparisons.

## 4. Conclusions

This is the first report of in vivo anti-cryptococcal activity of PPD isolated from *P. glandulosa*. The results indicate that i.p. administration of PPD against *C. neoformans* infection showed better efficacy at lower doses. In addition, no signs of discomfort were observed in the mice who were treated with PPD, which was administered either once or twice a day. Higher doses of PPD were equally effective when they were given orally.

## Figures and Tables

**Table 1:** Mean body weights of the mice injected i.p. with different doses of prosopilosidine (PPD). All values are expressed as mean ± SD (*n* = 5).

Dose of 1	Mean Body Weight of Mice on Day					
	1	2	3	4	5	6
Vehicle	24.40 ± 1.36	24.58 ± 1.4	24.37 ± 1.37	24.47 ± 1.41	24.55 ± 1.39	25.28 ± 0.88
1.0 mg/kg	24.83± 1.79	24.79 ± 1.81	23.66 ± 1.02	23.81 ± 0.95	24.28 ± 0.96	24.39 ± 0.93
2.5 mg/kg	24.8 ± 0.58	25.01 ± 0.68	25.1 ± 0.62	24.89 ± 0.37	24.85 ± 0.29	24.94 ± 0.28
5.0 mg/kg	23.55 ± 0.77	-	-	-	-	-
10 mg/kg	25.12 ± 0.66	-	-	-	-	-

**Table 2:** Blood chemistry profile of mice administered Prosopilosidine at 20 mg/kg via the oral route. All values are expressed as mean ± SEM (*n* = 3).

Parameters	Vehicle	Prosopilosidine (20 mg/kg)
Albumin (g/dL)	3.5 ± 0	3.1 ± 0
Alanine transaminase (ALT) U/L	35.0 ± 0	54.5 ± 26.5
Total bilirubin (mg/dL)	0.3 ± 0	0.2 ± 0
Blood urea nitrogen (BUN) mg/dL	21.0 ± 1	17.5 ± 0.5
Creatinine (CRE) mg/dL	0.245 ± 0.05	0.3 ± 0
BUN/CRE	89.3 ± 15.97	58.33 ± 1.67
Calcium (mg/dL)	10.65 ± 0.05	10.0 ± 0.3
Phosphate (mg/dL)	7.3 ± 0.1	9.5 ± 0
Glucose (mg/dL)	70.0 ± 9	145.5 ± 29.5
Na^+^ (mmol/L)	156.5 ± 2.5	150.5 ± 0.5
K^+^ (mmol/L)	8.4 ± 0.2	5.45 ± 0.25
Total protein (g/dL)	5.4 ± 0	5.0 ± 0
Globulin (g/dL)	1.95 ± 0.05	1.9 ± 0
